# Knowledge, Attitude, and Practices of Antibiotics and Antibiotic Resistance Among Chinese Pharmacy Customers: A Multicenter Survey Study

**DOI:** 10.3390/antibiotics9040184

**Published:** 2020-04-16

**Authors:** Pengchao Li, Khezar Hayat, Li Shi, Krizzia Lambojon, Amna Saeed, Muhammad Majid Aziz, Tao Liu, Shiyu Ji, Yilin Gong, Zhitong Feng, Minghuan Jiang, Wenjing Ji, Caijun Yang, Jie Chang, Yu Fang

**Affiliations:** 1 Department of Pharmacy Administration and Clinical Pharmacy, School of Pharmacy, Xi’an Jiaotong University, Xi’an 710061, China; lipengchao1996@stu.xjtu.edu.cn (P.L.); khezar.hayat@uvas.edu.pk (K.H.); 15802421326@163.com (L.S.); krizlambojon@gmail.com (K.L.); dr.amnasaeed92@gmail.com (A.S.); pharmajid82@yahoo.com (M.M.A.); liutao1031@stu.xjtu.edu.cn (T.L.); shishiyuyu07@stu.xjtu.edu.cn (S.J.); xjtugongyilin@163.com (Y.G.); fzt5051445@163.com (Z.F.); jiangmh2017@xjtu.edu.cn (M.J.); yfyx_8312@163.com (W.J.); yangcj@xjtu.edu.cn (C.Y.); jiechang@xjtu.edu.cn (J.C.); 2 Center for Drug Safety and Policy Research, Xi’an Jiaotong University, Xi’an 710061, China; 3 Shaanxi Centre for Health Reform and Development Research, Xi’an 710061, China; 4 Research Institute for Drug Safety and Monitoring, Institute of Pharmaceutical Science and Technology, China’s Western Technological Innovation Harbor, Xi’an 710061, China; 5 Institute of Pharmaceutical Sciences, University of Veterinary and Animal Sciences, Lahore 54000, Pakistan

**Keywords:** antibiotics, antibiotic resistance, knowledge, community pharmacy, China

## Abstract

Background: Resistance to antibiotics is one of the major global health challenges. An adequate understanding of the public regarding rational antibiotic use is a prerequisite to limit progression in antibiotic resistance. This study aimed to investigate the knowledge, attitude, and practices (KAP) of antibiotics and antibiotic resistance among customers visiting community pharmacies. Methods: This study was undertaken in three capital cities in China during March 2019 and July 2019 by using a questionnaire of 28 items. The questionnaire had four parts, including sociodemographic characteristics, KAP about antibiotics, and antibiotic resistance. A systematic random sampling approach was used to recruit the participants. Kruskal–Wallis and Mann–Whitney tests were carried out for data analysis. Results: The response rate was 66.7% (1800/2700). Out of the total, only 9.7% of the customers had good knowledge about antibiotics. Nearly half of the participants were unable to differentiate between antibiotics, and anti-inflammatory drugs (*n* = 820, 45.6%, Median = 2, IQR = 1). Most of the customers were of the view that the use of over the counter antibiotics in pregnant women is unsafe (*n* = 1307, 72.6%, Median = 2, IQR = 0). Almost half of the participants disagreed that costly antibiotics are more effective and have fewer side effects (*n* = 897, 49.9%, Median = 3, IQR = 1). Only 22.3% of participants said that they always finish the course of antibiotic treatment (*n* = 401, 22.3%, Median = 3, IQR = 1). Conclusion: The knowledge of Chinese pharmacy consumers was inadequate, and a lack of good attitudes and practices in certain aspects of antibiotic use was observed. Educational interventions are needed to increase public knowledge of antibiotics.

## 1. Introduction

Antibiotic resistance (ABR) is regarded as an important threat to the clinical effectiveness of antibiotics and is a major public health problem of global concern [1,2,3]. ABR has drastically amplified due to the irrational prescribing and/or inappropriate use of antibiotics [4,5]. There is a higher number of infections and outbreaks due to *Methicillin-resistant Staphylococcus aureus* (MRSA) and *Vancomycin-resistant enterococci* (VRE) since 2010 [6]. Parallel to the ABR problem, there have been less fruitful research and development in discovering the new antibiotics. Moreover, several million people die due to resistant infections worldwide annually [1]. The cost to manage these resistance infections has also significantly enhanced [7].

The unjudicial use of antibiotics is common in both hospital and community settings [8]. According to the World Health Organization (WHO), more than 60% of the total antibiotics are used in the community, and nearly half of them are misused [9]. There is also a direct relationship between the use of antibiotics and the emergence of ABR [10].

Just like other developing countries [4], China is also facing the problem of ABR [11]. China has been placed in the second spot in terms of the overall antibiotic consumption across all countries [12]. Recently, a study highlighted the prescribing of antibiotics in 80% of Chinese patients suffering from upper respiratory tract infections who visited the outpatient department (OPD) [13]. Additionally, a significant increase in resistance has also been noted against *Acinetobacter baumannii* and *carbapenem-resistant Klebsiella pneumonia* since 2005, as reported by the national resistance surveillance system of China [14]. Furthermore, the presence of colistin-resistant genes in community patients has further worsened the situation of ABR [15].

The sale of antibiotics without a prescription is not permitted in China as per the regulations of the China Food and Drug Administration in 2004 [16]. However, despite these regulations, the use of antibiotics without a prescription is a common practice across the country. A local study conducted in three different provinces of China showed high rates of dispensing antibiotics without a prescription from more than 50% of community pharmacies [17].

Several factors that could augment the irrational antibiotic prescribing among prescribers include patient demands, improper diagnosis, insufficient knowledge, and financial benefits [18].

Public education is one of the key interventions proposed by the WHO to rationalize the use of medicines [19]. Moreover, the improvement in public awareness and understanding of issues related to antibiotics is the principal strategic objective of the WHO Global Action Plan on Antimicrobial Resistance [20]. A variation in public knowledge in different countries about antibiotics has been highlighted by many studies [21]. The level of resistance was found to be higher in countries where there is a lack of awareness about ABR, such as in Sweden and the Netherlands, the knowledge of population is higher, and the ABR is lower [22]. It is believed that the perception of people towards medicines may affect their understanding of antibiotics and ABR [22,23,24]. Therefore, this study was aimed at determining the knowledge, attitude, and practices of customers about antibiotics and ABR visiting community pharmacies.

## 2. Methodology

This was a multicentered, survey-based study conducted in three capital cities of China, including Xi’an (western part), Nanjing (eastern part), and Changsha (central part) between April 2019 to August 2019. These cities were selected because they have a difference in socioeconomic and GDP level, such as the Xi’an has the lowest GDP per capita, whereas Nanjing has the highest GDP [25,26,27].

### 2.1. Survey Instrument

A thorough literature review was undertaken to conceptualize the questionnaire [28,29,30,31,32]. The initial version of the questionnaire was subjected to content and face validity by a team of experts (2 professors of pharmacy practice background and three customers). Some changes were made as per the feedback and opinions of the experts as few questions were redundant and difficult to understand, so they were modified or removed. Field tests on a small population comprised of 10 customers were also conducted (data were excluded).

The final version of the questionnaire had 28-items with four sections (see Supplementary File). The first section was comprised of 8 questions pointed towards demographic information, such as gender, age, education, monthly income, employment status, and purpose of the visit of the participants. In the second section, seven questions were asked to determine the antibiotic-related knowledge of the participants with options ‘yes,’ ‘no,’ and ‘unclear.’ The third section had eight questions related to attitude about antibiotics rated on the Likert scale, such as ‘strongly agree,’ ‘agree,’ ‘neutral,’ ‘disagree,’ and ‘strongly disagree.’ The last section had five questions focused on practices about antibiotics with five options ‘always,’ ‘often,’ ‘sometimes,’ ‘seldom,’ and ‘never.’ The overall scoring of knowledge, attitude, and practices were divided into three categories. A participant was found to have poor, average, or good knowledge if his score is <3, 3–5, or 6–7, respectively. Similarly, a participant was assigned to have a poor, average, or good attitude if his score is <14, 14–27, or 28–40. Additionally, a participant with a score, <9, 9–16, or 17–25, was found to have poor, average, or good practices.

The reliability of the questionnaire was determined by pre-testing the questionnaire on 20 customers (data excluded). The values of Cronbach’s α for the knowledge, attitude, and practice section were 0.76, 0.79, and 0.79, respectively, indicating an acceptable level of reliability.

### 2.2. Sampling

As of 2018, there are more than 0.45 million community pharmacies in China, which are categorized into large size (≥100 m^2^), medium size (50–100 m^2^), and small size (<50 m^2^). However, the large pharmacies were chosen as the survey field in this study due to their large client base (more than 500 customers per day). The information about community pharmacies and customer flow was obtained from official websites of local government [33,34,35]. With consideration of the density of the city’s population and survey operability, five large community pharmacies were selected from different main urban districts in each city eventually. The customers visiting these community pharmacies were recruited by the systematic random method (every 5th participant was selected) upon their entry to the pharmacy. Once they consented, they were interviewed by data collectors. Moreover, the participants having age less than 18 years and those who were buying cosmetics were excluded.

### 2.3. Data Collection

Trained data collectors were used to collect data. They were briefed about the aims and objectives of the study by the principal investigator. They approached pharmacy customers in three cities and administered the questionnaire. To maintain the quality and integrity of the data, a leader of the team visited different data collection sites randomly. Each questionnaire was double-checked to confirm whether the respondents have answered all of the survey items.

### 2.4. Data Analysis

The descriptive statistical analysis was carried to determine the frequencies and percentages of the data. Kolmogorov–Smirnov and Shapiro–Wilks tests were carried out to check the normality. Median knowledge, attitude, and practice scores were calculated, which were compared with the demographic variable by using Kruskal–Wallis and Mann–Whitney U tests. A *p*-value of less than 0.05 was considered to be statistically significant. Moreover, the relationship between scores of knowledge, attitude, and practices was evaluated using Spearman’s rank correlation test (*p* < 0.01). All statistical analyses were conducted using SPSS v.16.

### 2.5. Ethics Approval and Consent to Participate

The Biomedical Ethics Committee approved the study of Xi’an Jiaotong University, China (No. 2019-1030). Before starting the study, a brief introduction was provided to all the participants. Verbal consent was taken from all the participants before starting the questionnaire.

## 3. Results

**Figure 1:**
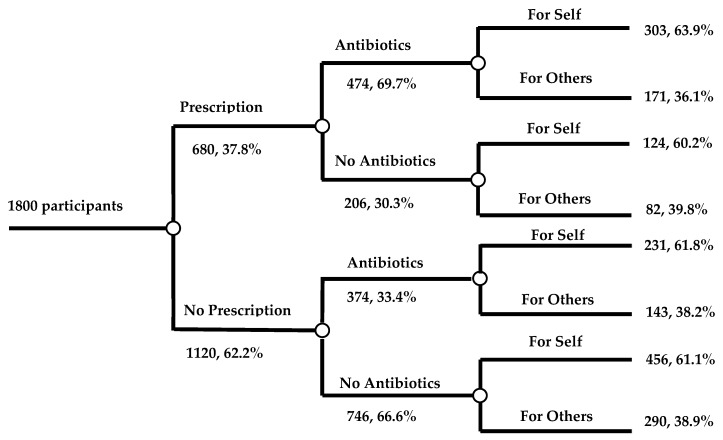
Purpose of customers visiting the community pharmacies.

Out of the total, one thousand and eight hundred customers in different cities who were visiting the community pharmacies to purchase medicine participated in this survey, including 58.8% (*n* = 1059) males and 41.2% (*n* = 741) females (Table 1). The proportion of participants of 26–35 years old (586, 32.6%) was larger than other age groups. Most of the customers (1201, 66.7%) had a college degree, and had a monthly income of 3000–8000 RMB (55.4% 998). Nearly half of the customers (44.1%) purchased antibiotics without a prescription pertaining to this visit. The customers purchased most of the antibiotics for themselves (63.0%). Figure 1 shows the purpose of all customers visiting the pharmacies.

### 3.1. Knowledge about Antibiotics

The knowledge of most of the participants was poor (22.3%) or average (69.4%); however, only 9.7% had good knowledge about antibiotics (Table 2). Nearly half of the participants thought antibiotics and anti-inflammatory medicines are the same (*n* = 820, 45.6%, Median = 2, IQR = 1); more than 70% of customers considered that antibiotics could be used to treat bacterial diseases (*n* = 1342, 74.6%, Median = 1, IQR = 1). More than half of respondents deemed antibiotics can be used to treat common cold (*n* = 949, 52.7%, Median = 1, IQR = 1).

Almost one-fifth of the participants thought that antibiotics would not kill normal flora (*n* = 403, 22.4%, Median = 2, IQR = 0). A large number of participants supposed that the unnecessary use of antibiotics is dangerous for health (*n* = 1396, 77.5%, Median = 1, IQR = 0). The majority of the customers were aware that the use of over-the-counter (OTC) antibiotics in pregnant women is unsafe (*n* = 1307, 72.6%, Median = 2, IQR = 0). Only less than 40% of customers held that antibiotics could not be used along with traditional Chinese medicines (*n* = 676, 37.6%, Median = 2, IQR = 1).

### 3.2. Attitude about Antibiotics

The attitude of most of the participants (55.5%) was good (Table 3). Almost half of the participants disagreed that costly antibiotics are more effective and have fewer side effects (*n* = 897, 49.9%, Median = 3, IQR = 1). Less than half of the participants disagreed that expensive antibiotics have fewer side effects (*n* = 868, 48.2%, Median = 3, IQR = 1). Most of the participants agreed that antibiotic use without a doctor’s prescription is unsafe (*n* = 1153, 63.1%, Median = 4, IQR = 1). More than 70% of respondents disagreed that taking a double dose of antibiotics can speed up the cure of disease (*n* = 1301, 72.3%, Median = 4, IQR = 1). More than half of the participants agreed that taking multiple antibiotics are unable to produce desired results compared to a single antibiotic (*n* = 1146, 63.7%, Median = 4, IQR = 1). More than half of the participants were of the view that the effectiveness of treatment would be reduced if a full course of antibiotics is not completed (*n* = 1044, 58.0%, Median = 2, IQR = 1). Less than half of the participants disagreed that it is better to stop taking antibiotics when symptoms are improved (*n* = 848, 47.1%, Median = 3, IQR = 2). Almost 45% of participants agreed that leftover antibiotics could not be saved and used for the same symptoms again (*n* = 804, 44.6%, Median = 3, IQR = 1).

### 3.3. Practice about Antibiotics

Nearly seventy percent of the participants (69.3%) showed a good attitude towards antibiotic use (Table 4). Only 4.2% of participants never read the instructions about the use of antibiotics carefully before taking them (*n* = 75, 4.2%, Median = 2, IQR = 2). Only 22.3% of participants always finish the course of antibiotic treatment (*n* = 401, 22.3%, Median = 3, IQR = 1). Almost one-third of participants never change the dose during antibiotic treatment (*n* = 592, 32.9%, Median = 4, IQR = 2). More than half of the participants agreed that they switch antibiotics during treatment (*n* = 1072, 59.6%, Median = 4, IQR = 2). The majority of participants kept leftover antibiotics at home in case of future need (*n* = 1530, 85.0%, Median = 3, IQR = 2).

### 3.4. Association of Median Scores of Participants with Demographic Variables

Table 5 illustrates the relationship between median scores with demographic variables. The knowledge scores of different ages, education, income, and employment have significant differences (*p* < 0.05). Moreover, the median attitude score was significantly associated with gender, age, education, and employment. However, the practice scores were not significantly associated with demographic variables.

The correlation analysis revealed a positive correlation between knowledge, attitude, and practice, which means knowledge affects attitude and practice. Additionally, the attitude has also an influence on practice (Table 6). 

## 4. Discussion

ABR has attracted extensive attention to society due to its devastating effects on health. KAP study towards antibiotics helps realize the current perceptions of major stakeholders. To our knowledge, this study is the first of its kind to investigate the knowledge, attitudes, and practice of pharmacy customers about antibiotics in China.

Our findings have shown that the knowledge of antibiotics was not good in customers visiting different community pharmacies of China, and they were unaware of several aspects of antibiotics. For example, 45.6% of customers considered antibiotics and anti-inflammatory medicines as the same drugs, and 52.7% believed that common cold could be treated with an antibiotic. These findings are similar to a systematic review, which showed that 50.9% of the participants were unaware of the difference between antibiotics and anti-inflammatory drugs [36]. Moreover, half of the participants in Awad’s study conducted in Kuwait thought that antibiotics could treat the common cold [37].

The knowledge of the participants in our study was higher compared to a previous Chinese survey conducted in 2015 [33]. Higher knowledge could be explained that pharmacy customers were more concerned about their use of antibiotics as these drugs are to be used by themselves or their beloved ones. Moreover, doctors and pharmacists-led campaigns focused on the rational use of antibiotics have also improved the understanding of the public toward prudent antibiotic use [28,38,39]. However, the knowledge level of our study participants was lower than studies conducted in European countries [24,28] because of the difference in economic development and health literacy among people.

Pharmacists in community pharmacies, who provide consultative services to the customers, play a vital role in the rational use of antibiotics. A recent Chinese multicenter cross-sectional study [40] showed that 45.9% of pharmacists did not know that antibiotics are not effective in viral infections. Several studies [41,42,43] revealed that pharmacist-led educational interventions could improve public or pharmacy customers’ knowledge about the rational use of antibiotics. Community pharmacists should have higher knowledge, which could help enhance antibiotics knowledge of pharmacy customers gradually. Healthcare professionals, including physicians and pharmacists, who are responsible for the rational use of antibiotics, should monitor patients having an antibiotic prescription and should promote the counseling of customers to help them better understand antibiotics and antibiotic resistance [38,39].

It was noted that the participants having higher education had better antibiotic-related knowledge, as also highlighted by Grosso’s study [44]. Moreover, young people can quickly get information about the antibiotic from television, the internet, and other channels, including social media, due to ease in accessibility. However, older people are more likely to rely on their own experience for the use of antibiotics [45].

Our findings showed that attitudes of participants to antibiotics were inappropriate in some aspects, as 35.9% of customers were familiar that antibiotics could not be used without a doctor’s prescription. Almost half of the participants agreed that “when symptoms are improved, you can stop using antibiotics.” Knowledge has a strong effect on attitude [46], so these attitudes towards antibiotics may be driven from poor knowledge.

The present study illustrated that the habits of antibiotics usage were not good enough as 85.0% of respondents keep leftover antibiotics at home in case of future need, which could trigger the self-medication of antibiotics, thereby impacting the AMR burden [47]. The rate of antibiotic self-medication was 20.8%, which is in line with a study conducted in Cameroon [48]. However, the rate of self-medication in our study was lower than the previous Chinese study, which reported self-medication among 29.4% enrolled participants [49]. This may be due to the difference in the study population and the survey instrument.

Nearly half (44.1%) of the pharmacy customers purchased antibiotics without prescriptions during this survey, which is low compared to a recent study conducted in China (70.1%), possibly due to the difference in the survey method [50]. Jie Chang surveyed the dispensing of antibiotics in 1690 community pharmacies by using a simulated client method. However, we conducted a KAP survey of pharmacy customers visiting 15 large pharmacies in an urban area. Therefore, it is needed that the drug regulatory department and the community pharmacy should strengthen the control on the sale of antibiotics.

There are several limitations to this study. First, community pharmacies were chosen from the urban area, so it only reflects the situation of the metropolitan area; however, the view of community pharmacists working in the rural area of China may differ. Second, the study is cross-sectional, so it may not mirror any dynamic changes in the KAP of customers. Third, self-medication to antibiotics was only considered from a prescription without including the use of left-over antibiotics at home. Regardless of the above limitations, our study provides useful insight into the understanding of antibiotics among pharmacy customers in China.

## 5. Conclusions

An inadequate level of knowledge about antibiotics was found among Chinese pharmacy consumers. Moreover, their attitudes and practices toward antibiotic use were not good in several aspects. The government should take some measures or interventions, including mass level education, about the appropriate use of antibiotics to improve the knowledge and rational use of antibiotics among the public.

## Figures and Tables

**Table 1:** Demographic information of customers (*n* = 1800).

Variable	Frequency (n)	Percentage (%)
**Gender**		
Male	1059	58.8
Female	741	41.2
**Age (years)**		
18–25	404	22.4
26–35	586	32.6
36–45	438	24.3
46–59	291	16.2
>60	81	4.5
**Education**		
No formal education	21	1.2
Primary school	47	2.6
Junior high school	168	9.3
Senior high school	363	20.2
College and above	1201	66.7
**Income (RMB)**		
<1000	218	12.1
1000–3000	326	18.1
3001–5000	587	32.6
5001–8000	411	22.9
>8000	258	14.3
**Employment**		
Employed	1091	60.7
Self-employed	258	14.3
Unemployed	112	6.2
Student	229	12.7
Other	110	6.1

**Table 2:** Knowledge of participants about antibiotics (*n* = 1800).

Question (Correct Answer)	Yes	No	Unclear	Median (IQR)
Antibiotics and anti-inflammatory medicines are the same (no)	820 (45.6)	642 (35.7)	338 (18.7)	2 (1)
Antibiotic can be used to treat bacterial diseases, i.e., pneumonia, typhoid, and wound infections (yes)	1342 (74.6)	161 (8.9)	297 (16.5)	1 (1)
Antibiotic can be used to treat common cold (no)	949 (52.7)	559 (31.1)	292 (16.2)	1 (1)
The antibiotics will not kill normal flora (no)	403 (22.4)	970 (53.9)	427 (23.7)	2 (0)
Unnecessary use of antibiotics is dangerous for health (yes)	1396 (77.5)	176 (9.8)	228 (12.7)	1 (0)
OTC use of antibiotics in pregnant women is safe (no)	127 (7.1)	1307 (72.6)	366 (20.3)	2 (0)
Antibiotics can be used along with tradition Chinese medicines (yes)	319 (17.7)	676 (37.6)	805 (44.7)	2 (1)

**Table 3:** Attitude of participants about antibiotics (*n* = 1800).

Question	1-Strongly Agree; 5-Strongly Disagree. N (%)					Median (IQR)
	SA	A	N	D	SD	
Costly antibiotics are more effective and have fewer side effects.	95 (5.3)	226 (12.6)	582 (32.3)	707 (39.3)	190 (10.5)	3 (1)
Costly antibiotics have fewer side effects.	68 (3.8)	247 (13.7)	617 (34.3)	695 (38.6)	173 (9.6)	3 (1)
Antibiotic use without a doctor’s prescription is safe.	51 (2.8)	248 (13.8)	348 (19.3)	824 (45.8)	329 (18.3)	4 (1)
Taking double dose of antibiotics can speed up the cure of diseases.	48 (2.7)	127 (7.0)	324 (18.0)	864 (48.0)	437 (24.3)	4 (1)
Taking many antibiotics produce the better result than one antibiotic.	53 (2.9)	164 (9.1)	437 (24.3)	784 (43.6)	362 (20.1)	4 (1)
The effectiveness of treatment would be reduced if a full course of antibiotics was not completed.	185 (10.3)	859 (47.7)	441 (24.5)	225 (12.5)	90 (5.0)	2 (1)
It is better to stop taking antibiotic when symptoms are improved.	81 (4.5)	386 (21.4)	485 (27.0)	693 (38.5)	155 (8.6)	3 (2)
The leftover antibiotics can be saved and used for the same symptoms again.	83 (4.6)	360 (20.0)	553 (30.7)	627 (34.9)	177 (9.8)	3 (1)

SA: strongly agree, A: agree, N: neutral, D: disagree, SD: strongly disagree.

**Table 4:** Practices of participants about antibiotics (*n* = 1800).

Question	Always N (%)	Often N (%)	Sometimes N (%)	Seldom N (%)	Never N (%)	Median (IQR)
I read the instructions in the package insert carefully before taking antibiotics.	654 (36.3)	563 (31.3)	294 (16.3)	214 (11.9)	75 (4.2)	2 (2)
I finish the full course of antibiotic treatment.	401 (22.3)	452 (25.1)	572 (31.8)	281 (15.6)	94 (5.2)	3 (1)
I change the dose during antibiotic treatment.	68 (3.8)	123 (6.8)	501(27.8)	516 (28.7)	592 (32.9)	4 (2)
I switch antibiotics during the course of treatment.	66 (3.7)	99 (5.5)	415 (23.1)	492 (27.3)	728 (40.4)	4 (2)
I keep leftover antibiotics at home in case of future need.	294 (16.4)	395 (21.9)	436 (24.2)	405 (22.5)	270 (15.0)	3 (2)

**Table 5:** Median score association with demographics (*n* = 1800).

Variable	Median Knowledge Score (IQR)	*p*-Value	Median Attitude Score (IQR)	*p*-Value	Median Practice Score (IQR)	*p*-Value
**Gender**						
Male	2.00(1.00)	0.28	3.50(1.00)	<0.001	3.00(2.00)	0.58
Female	2.00(1.00)		3.50(1.00)		3.00(1.00)	
**Age (years)**						
18–25	2.00(1.00)	0.002	3.50(1.00)	<0.001	3.00(2.00)	0.483
26–35	2.00(1.00)		3.50(1.00)		3.00(1.00)	
36–45	2.00(1.00)		3.50(1.00)		3.00(1.00)	
46–59	2.00(1.00)		3.50(1.00)		3.00(1.00)	
>60	2.00(1.50)		3.00(1.00)		3.00(2.00)	
**Education**						
Out of School	3.00(2.00)	<0.001	3.00(2.25)	<0.001	3.00(4.00)	0.561
Primary school	2.00(2.00)		3.00(1.00)		3.00(1.00)	
Junior high school	2.00(2.00)		3.00(1.00)		3.00(1.75)	
Senior high school	2.00(1.00)		3.50(1.00)		3.00(1.00)	
College and above	2.00(1.00)		3.50(1.00)		3.00(1.00)	
**Income (RMB)**						
<1000	2.00(0.25)	0.003	3.50(1.00)	0.760	3.00(2.00)	0.161
1000–3000	2.00(1.00)		3.50(1.00)		3.00(2.00)	
3001–5000	2.00(1.00)		3.50(1.00)		3.00(1.00)	
5001–8000	2.00(1.00)		3.50(1.00)		3.00(1.00)	
>8000	2.00(1.00)		3.50(1.00)		3.00(2.00)	
**Employment**						
Employed	2.00(1.00)	0.018	3.50(1.00)	<0.001	3.00(1.00)	0.413
Self-employed	2.00(1.00)		3.50(1.00)		3.00(1.00)	
Unemployed	2.00(1.00)		3.00(1.00)		3.00(1.00)	
Student	2.00(0.00)		4.00(1.00)		3.00(2.00)	
Other	2.00(1.00)		4.00(1.00)		3.00(1.00)	

**Table 6:** Correlation analysis between knowledge score, attitude score, and practice score.

		Score	Knowledge	Attitude	Practice
	Coefficient				
Score					
Knowledge			1	0.299**	0.173**
Attitude			0.299**	1	0.252**
Practice			0.173**	0.252**	1

** means significance test *p* < 0.01. 0–0.09, no correlation; 0.1–0.3, weak correlation; 0.3–0.5, medium correlation; 0.5–1.0, strong correlation.

## Supplementary Materials

The following are available online at https://www.mdpi.com/2079-6382/9/4/184/s1, Supplementary Files: Knowledge, attitude and practices of pharmacy customers about antibiotics
