# Supplementary material for: Knowledge, Attitude, and Practices of Antibiotics and Antibiotic Resistance Among Chinese Pharmacy Customers: A Multicenter Survey Study
Source: Antibiotics (Basel). 2020 Apr 16;9(4):184. doi: 10.3390/antibiotics9040184 (PMC7235738; doi:10.3390/antibiotics9040184)
Supplement: Supplementary file 1 [file antibiotics-09-00184-s001.pdf]

Supplementary File: Knowledge, attitude and practices of pharmacy customers about antibiotics

Section A: Personal Information

Gender	☐Male ☐Female
Age Group	☐18-25 ☐26-35 ☐36-45 ☐46-59 ☐>60
Educational Degree	☐ No formal education ☐ Primary school ☐ Junior high school ☐ Senior high school ☐ College and above
Monthly income	☐<1000 ☐1000-3000 ☐3000-5000 ☐5000-8000 ☐>8000
Employment	☐ Employed ☐ Self-employed ☐ Unemployed ☐ Student ☐ Others
Do you have prescription in this purchasing?	☐ Yes ☐ No
Do you have antibiotics in this purchasing?	☐ Yes ☐ No
Who do you have antibiotics for in this purchasing?	☐ Children or teenagers ☐ For yourself ☐ For others

Section B: Knowledge

Question	Yes	No	Not clear
1. Antibiotics and anti–inflammatory medicines are the same.	☐	☐	☐
2.Antibiotic can be used to treat bacterial diseases i.e. pneumonia, typhoid and wound infections.	☐	☐	☐
3. Antibiotic can be used to treat common cold.	☐	☐	☐
4. The antibiotics will not kill normal flora.	☐	☐	☐
5. Unnecessary use of antibiotics is dangerous for health.	☐	☐	☐
6. Use of antibiotics in pregnant women is safe.	☐	☐	☐

7.Antibiotics can be used along with tradition Chinese medicines	☐	☐	☐

Section C: Attitude

Question	1-strongly agree；5-not strongly disagree				
	SA	A	N	D	SD
1. Do you think costly antibiotics are more effective.	☐	☐	☐	☐	☐
2. Do you think costly antibiotics have fewer side effects.	☐	☐	☐	☐	☐
3. Do you think using antibiotic without a doctor's prescription is safe?	☐	☐	☐	☐	☐
4. Do you think using double dose of antibiotics can speed up the cure of a diseases.	☐	☐	☐	☐	☐
5. Do you think using many antibiotics produce the better result than one antibiotic.	☐	☐	☐	☐	☐
6. Do you agree the effectiveness of treatment would be reduced if a full course of antibiotic was not completed	☐	☐	☐	☐	☐
7. Do you think it better to stop taking antibiotic when symptoms are improved.	☐	☐	☐	☐	☐
8. Do you think the leftover antibiotics can be saved and used for the same symptoms again.?	☐	☐	☐	☐	☐

Section D: Practices

Question	always	often	sometimes	seldom	never
1. How often do you read the instruction in the package insert carefully before taking antibiotics?	☐	☐	☐	☐	☐
2. How often do you finish the full course of antibiotic treatment?	☐	☐	☐	☐	☐
3. Do you change the dose during the course of antibiotic treatment?	☐	☐	☐	☐	☐

4. How often do you switch antibiotics during the course of treatment?	☐	☐	☐	☐	☐
5. How often do you keep leftover antibiotics at home in case of future need?	☐	☐	☐	☐	☐

Thank you very much for your time
